# Risk factors for pulmonary tuberculosis in patients with chronic obstructive airway disease in Taiwan: a nationwide cohort study

**DOI:** 10.1186/1471-2334-13-194

**Published:** 2013-04-30

**Authors:** Chih-Hsin Lee, Ming-Chia Lee, Chin-Chung Shu, Chor-Shen Lim, Jann-Yuan Wang, Li-Na Lee, Kun-Mao Chao

**Affiliations:** 1 Graduate Institute of Biomedical Electronics and Bioinformatics, National Taiwan University, No. 1, Roosevelt Road, Sec. 4, Taipei 10617, Taiwan; 2 Department of Internal Medicine, Buddhist Tzu Chi General Hospital, Taipei, Taiwan; 3 School of Medicine, Tzu Chi University, No.701, Zhongyang Road, Sec. 3, Hualien 97004, Taiwan; 4 Department of Pharmacy, Buddhist Tzu Chi General Hospital, Taipei Branch, 289, Jianguo Road, Xindian District, New Taipei City 23142, Taiwan; 5 Department of Traumatology, National Taiwan University Hospital, Taipei, Taiwan; 6 Department of Internal Medicine, National Taiwan University Hospital, Taipei, Taiwan; 7 Department of Laboratory Medicine, National Taiwan University Hospital, No. 7, Chung-Shan South Road, Taipei 100, Taiwan

**Keywords:** Chronic obstructive pulmonary disease, Tuberculosis, Inhaled corticosteroid, Oral corticosteroid, Time-dependent cox regression

## Abstract

**Background:**

An association between chronic obstructive pulmonary disease (COPD) and tuberculosis (TB) has been described, mainly due to smoking and corticosteroid use. Whether inhaled corticosteroid (ICS) therapy is associated with an increased risk of TB remains unclear.

**Methods:**

We selected COPD cases by using six diagnostic scenarios and control subjects from a nationwide health insurance database, and applied time-dependent Cox regression analysis to identify the risk factors for TB.

**Results:**

Among 1,000,000 beneficiaries, 23,594 COPD cases and 47,188 non-COPD control subjects were selected. Cox regression analysis revealed that age, male gender, diabetes mellitus, end-stage renal disease, and cirrhosis, as well as COPD (hazard ratio = 2.468 [2.205–2.762]) were independent risk factors for TB. Among the COPD cases, those who developed TB received more oral corticosteroids and oral β-agonists. Time-dependent Cox regression analysis revealed that age, male gender, diabetes mellitus, low income, oral corticosteroid dose, and oral β-agonist dose, but not ICS dose, were independent risk factors for TB. The identified risk factors and their hazard ratios were similar among the COPD cases selected using different scenarios.

**Conclusion:**

Keeping a high suspicion and regularly monitoring for the development of pulmonary TB in COPD patients are necessary, especially for those receiving higher doses of oral corticosteroids and other COPD medications. Although ICS therapy has been shown to predispose COPD patients to pneumonia in large randomized clinical trials, it does not increase the risk of TB in real world practice.

## Background

Tuberculosis (TB) is a major cause of death worldwide. The World Health Organization estimated that there were 8.7 million new cases of TB in 2011 [1]. The risk factors for TB included age, male gender, low socioeconomic status, malnutrition, substance abuse, silicosis, human immunodeficiency virus infection, malignancy, diabetes, renal disease, celiac disease, gastrectomy, transplant, and receiving corticosteroids and tumor necrosis factor inhibitors [2-7]. In addition, an association between obstructive pulmonary disorders (i.e. chronic obstructive pulmonary disease [COPD] and asthma) and active TB has been described, mainly due to smoking and corticosteroid use [7].

COPD is an airway inflammatory disease with a high prevalence rate worldwide [8], ranging from 8.2% in China, 10.9% in Japan, 19.6% in USA, 23.8% in South Africa to 26.1% in Australia [9-13], It is a major health burden both in developed and developing countries. Inhaled corticosteroids (ICS), along with long-acting β-agonists (LABA), are currently recommended for patients with severe COPD with repeated exacerbations [8]. ICS therapy has been shown to predispose COPD patients to pneumonia in large randomized clinical trials [14,15]. Causative microorganisms of pneumonia, however, were not described. Although the systemic administration of corticosteroids is a known risk factor for TB [7], whether ICS therapy is associated with an increased risk of TB has yet to be elucidated.

A previous study on patients with COPD confirmed by pulmonary function testing suggested that the use of high-dose ICS, defined as an equivalent fluticasone dose >500 μg per day, is associated with the development of pulmonary TB in endemic areas of TB [16]. A nested case–control study using a cohort of patients with airways disease in the Quebec database also revealed that exposure to ICS was associated with an increased risk of TB in patients not receiving oral corticosteroids [17]. In both studies, the results of Cox regression analysis may not truly reflect the impact of ICS exposure, as time-dependent analysis for the variable intensity of exposure was not performed and the consumption of respiratory medications other than ICS and oral corticosteroids (OCS) was not considered. In addition, due to a lack of data on pulmonary function testing, the severity of airway obstruction was not determined and stratified in the latter study.

The National Health Insurance (NHI) program of Taiwan is a mandatory universal health insurance program, offering comprehensive medical care coverage to more than 95% of the residents of Taiwan since 1996 [18]. In this study, we used the NHI database and applied time-dependent Cox proportional hazards models to identify the risk factors for developing active pulmonary TB with a special emphasis on the influence of COPD and its medications.

## Methods

The Institutional Review Board of National Taiwan University Hospital, Taipei, Taiwan approved the study and waived the need for informed consent due to the retrospective design (NTUH IRB: 201106091RC). The study comprised of two parts. First, COPD cases and control subjects matched in age (within 5 years), sex and timing of entering the database were selected (1:2 in case numbers) from the Longitudinal Health Insurance Database (LHID) 2005, a subset database of the NHI program. The characteristics of the LHID 2005 have been described previously [19]. We studied the influence of COPD as well as other co-morbidities on the risk of developing TB. In the second part of the study, the effect of COPD medications on the risk of developing TB was investigated with a time-dependent approach among patients with COPD. All selected cases were followed until commencement of anti-TB treatment, December 31, 2007, or lost to follow-up (canceled health insurance prior to December 31, 2007).

### Selection criteria of COPD

**Figure 1:**
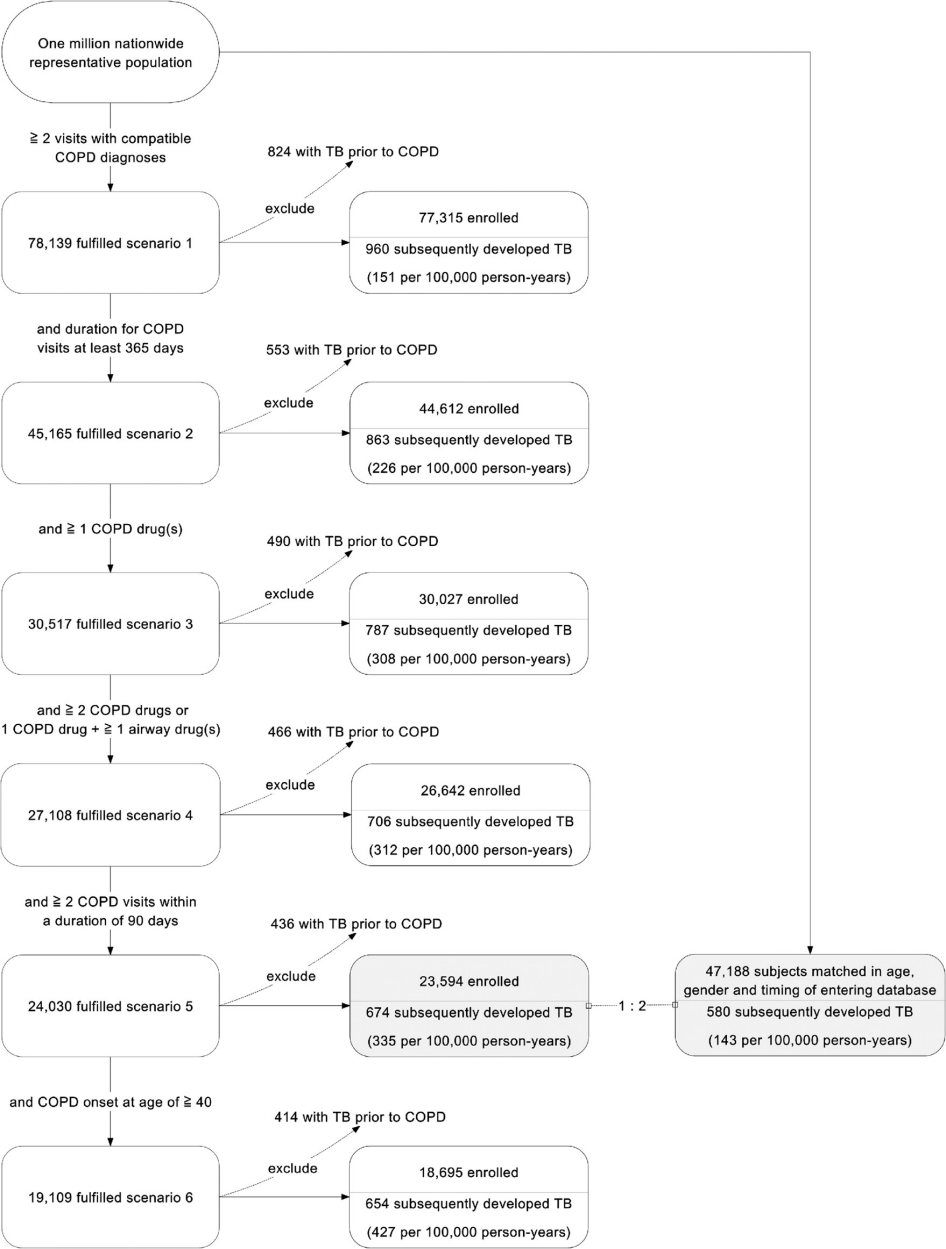
**Study flow chart.** The number of chronic obstructive pulmonary disease (COPD) cases identified under different selection scenarios and the number of subsequent tuberculosis (TB) cases.

Because of the wide spectrum of the clinical presentation of COPD, the ambulatory care and inpatient discharge records were analyzed to identify COPD patients from 1996 to 2007 using six different selection scenarios combining diagnostic code, duration and interval of medical visits with compatible diagnoses, prescriptions, and age of onset. The selection scenarios of COPD started from a loose criteria and gradually narrowed down to severe COPD requiring multiple medications and out-patient visits (scenarios 1 – 6 in Figure 1). The compatible COPD diagnoses included International Classification of Diseases, ninth revision, clinical modification (ICD-9-CM) codes 490–492, and 496, and A-code A323, A325. Prescriptions for COPD were defined as at least two COPD-specific medications or one COPD-specific medication plus at least one airway medication(s) during a period of 90 days. The COPD-specific medications included prescriptions containing corticosteroids (inhaled, oral or parenteral), β-agonists (long-acting or short-acting; inhaled or oral), anti-cholinergics (ipratropium or tiotropium), aminophylline, and theophylline. Airway medications included oral antitussives, mucolytic agents, and sympathomimetics.

Patients who developed active TB prior to or within 180 days following the onset of COPD were excluded. The number of selected COPD cases in each scenario and the subsequent events of active TB during follow-up are shown in Figure 1. The index date was defined as the date of first visit or admission with a compatible diagnosis for the COPD cases, and the corresponding date after entering the LHID 2005 database for the control subjects. COPD patients with an index date prior to January 1, 1997 were excluded to ensure an observation period of one year to monitor active TB treatment or diagnosis. Patients with a follow-up duration of less than six months after the index date were also excluded to ensure a sufficient observation period to confirm the subsequent development of TB.

Acute exacerbations (AEs) of COPD were defined as emergency department visits or admissions with compatible COPD diagnosis codes, plus a prescription of systemic corticosteroids. An exacerbation occurring within 7 days following a prior AE was considered as a continuation of the prior episode.

### Definition of active TB

**Figure 2:**
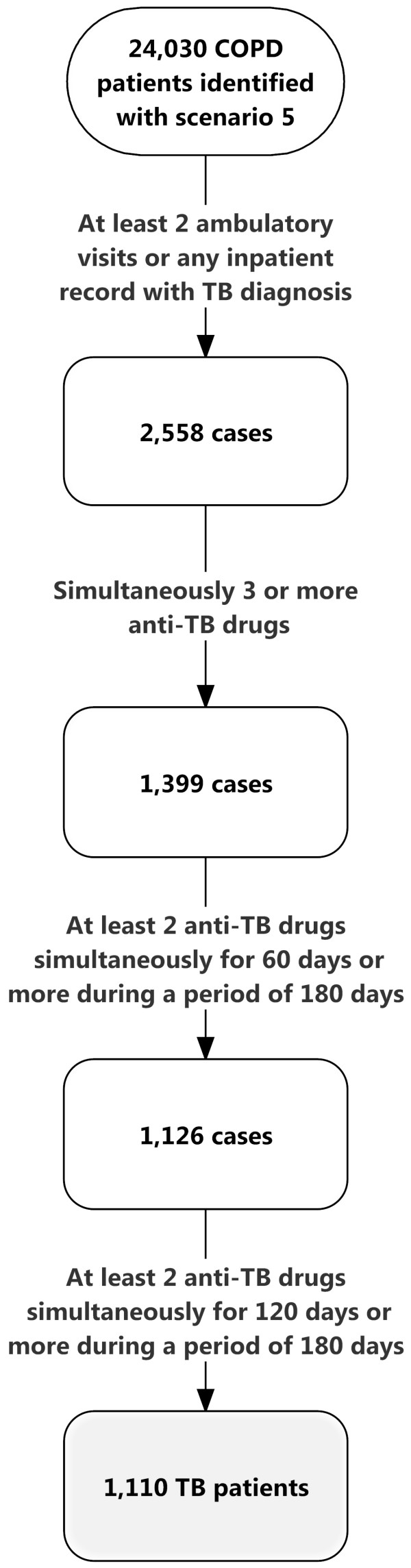
**Selection for TB patients.** The number of TB cases identified in each step among COPD patients selected by scenario 5.

Based on our previous publication [19], active TB was defined by at least two ambulatory visits or one inpatient record with a compatible diagnosis (ICD-9-CM codes 010–012, and 018, and A-codes A020, A021), plus at least one prescription consisting of three or more than three anti-tuberculous drugs. There should be prescriptions of at least two anti-tuberculous drugs simultaneously for 120 days or more during a period of 180 days. The anti-tuberculous drugs included isoniazid, rifampicin, ethambutol, pyrazinamide, prothionamide, terizidone, streptomycin, kanamycin, quinolones, cycloserine, and aminosalicylic acid. For patients with end-stage renal disease, the prescriptions were adjusted according to treatment guidelines [20]. The TB notification rates using these diagnostic criteria for each COPD selection scenario are shown in Figure 1. The number of TB cases identified in each step among COPD patients selected by scenario 5 was demonstrated in Figure 2.

### Comorbidities

Malignancy, diabetes mellitus, end-stage renal disease, pneumoconiosis, liver cirrhosis, autoimmune diseases, and acquired immunodeficiency disease were identified according to our previous publication [19]. The low income group was identified from the insurance status and required the annual household income to be below 4500 US dollars [21].

### Statistical analysis

The prescription duration of individual drugs was converted from the claims data according to the defined daily doses [22] and grouped according to their pharmacological categories. Inhaled and systemic corticosteroids were converted to equivalent doses of budesonide 800 micrograms [23] and prednisolone in grams [7], respectively.

Inter-group differences were compared using the independent samples *t*-test for numerical variables and chi-square test or Fisher’s exact test for categorical variables as appropriate. Curves of time to commencement of anti-TB treatment were generated using the Kaplan-Meier method and were compared using the log-rank test.

The effects of COPD and other co-morbidities on the risk of developing pulmonary TB were compared between the COPD patients and control subjects using the Cox proportional hazards model. Among the COPD patients, a time-dependent Cox proportional hazard model was applied to investigate the effect of co-morbidities and respiratory medications on the risk of developing pulmonary TB. The variables in the analysis included sex, age, the prescribed dose of each class of respiratory medication, and comorbidities. Each time-dependent drug prescription was determined as the total prescribed dose from 360 days to 90 days prior to each outcome event. An average dose of ICS ≥ budesonide 800 microgram per day during the period were defined as high-dose ICS.

The prevalence of smoking was much lower among female COPD patients (24.2%) than that among the general COPD patients (82.4%) in Taiwan [24,25]. To explore the confounding by smoking, sub-population analysis focusing on males and females separately was conducted.

Basic model-fitting techniques, including variable selection, goodness-of-fit, area under the receiver operating characteristic curve, adjusted generalized *R*^2^ and regression diagnostics (residual analysis, detection of influential cases, and check for multicollinearity), were applied to assure the quality of the multivariate analysis. Significance levels for entry and stay were set at 0.15. A two-sided *p* value of less than 0.05 was considered significant. All analyses were performed using SAS software (SAS Institute Inc., Cary, NC, USA).

## Results

**Figure 3:**
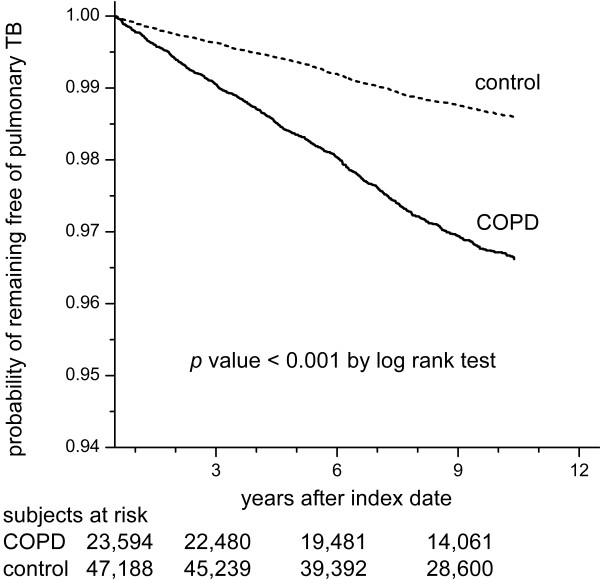
**Curves of time to commencement of anti-TB treatment.** The curves of time to commencement of anti-TB treatment generated by the Kaplan-Meier method among cases of chronic obstructive pulmonary disease (COPD) selected under scenario 5 and the control subjects.

Among the 1,000,000 beneficiaries in the LHID 2005, 23,594 COPD cases were identified, and 47,188 non-COPD subjects matched for age, gender, and timing of entering the LHID 2005 were selected as the control group. Their clinical characteristics are summarized in Table 1. The mean age of the COPD cases was 54.5 ± 22.9 years with a male–female ratio of 1.6. The COPD group had a significantly higher risk of developing TB than the control group (2.9% *vs*. 1.2%, *p* < 0.001 by the *chi*-square test), which also resulted in a shorter duration of follow-up for the COPD group. Curves of time to commencement of anti-TB treatment of the two groups were obvious separated as calculated by the log-rank test (Figure 3). The most common underlying comorbidities in the COPD group were diabetes mellitus and malignancy. The COPD subjects were more likely to have lung cancer and autoimmune diseases. Furthermore, more COPD subjects had a low income compared with the control subjects.

**Table 1:** Characteristics of the COPD patients selected under scenario 5 and the control subjects

	**COPD ** **(N = 23,594)**	**Control ** **(N = 47,188)**	***p *value**
Age (years)	54.5 ± 22.9	54.5 ± 22.9	
Male	14,635 (62.0)	29,270 (62.0)	
Developed TB	674 (2.9)	554 (1.2)	<0.001^#^
Follow-up duration (years)	8.63 ± 2.54	8.71 ± 2.47	<0.001*
Diabetes mellitus	1,573 (6.7)	3126 (6.6)	0.831^#^
Malignancy	337 (1.4)	634 (1.3)	0.361^#^
other cancer	300 (1.3)	619 (1.3)	0.656^#^
lung cancer	37 (0.2)	15 (0.0)	<0.001^#^
Autoimmune disease	65 (0.3)	61 (0.1)	<0.001^#^
End-stage renal disease	37 (0.2)	82 (0.2)	0.604^#^
Liver cirrhosis	8 (0.0)	10 (0.0)	0.317^#^
Transplantation	4 (0.0)	6 (0.0)	0.740^‡^
Pneumoconiosis	2 (0.0)	0 (0.0)	0.111^‡^
HIV/AIDS	0 (0)	3 (0)	0.556^‡^
Low income	340 (1.4)	391 (0.8)	<0.001^#^

HIV: human immunodeficiency virus, AIDS: acquired immunodeficiency syndrome;

Data are shown as mean ± SD or No. (%) unless otherwise indicated.

* calculated by the independent-samples *t* test.

^#^ calculated by the chi-square test.

^‡^ calculated by Fisher’s exact test.

Cox proportional hazards regression analysis including all of the variables listed in Table 1 for the 23,594 COPD cases and the 47,188 control subjects revealed that COPD was an independent risk factor for developing pulmonary TB (hazard ratio = 2.468 [2.205 – 2.762]) (Table 2). We also found that increased age, male gender, diabetes mellitus, end-stage renal disease, and liver cirrhosis significantly increased the risk of developing pulmonary TB. In the sub-population analyses focusing on males and females separately, COPD remained an independent risk factor associated with pulmonary TB in either genders with a similar hazard ratio (Table 3).

**Table 2:** Cox regression analysis for risk factors of pulmonary tuberculosis

**Variables**	***p*value**	**Hazard ratio**	**95% confidence interval**	
			**Lower**	**Upper**
Age (10 years)	<0.001	1.464	1.406	1.524
Male	<0.001	2.181	1.901	2.501
COPD	<0.001	2.468	2.205	2.762
Diabetes mellitus	0.001	1.491	1.242	1.790
End-stage renal disease	0.037	2.602	1.059	6.393
Liver cirrhosis	0.007	6.958	1.682	28.776

Cox regression analysis for factors possibly associated with the development of active tuberculosis in the 23,594 cases of chronic obstructive pulmonary disease (COPD) selected under scenario 5 and the 47,188 non-COPD controls.

**Table 3:** Cox regression analysis for the impact of chronic obstructive pulmonary disease (COPD) on risk of developing pulmonary tuberculosis in male and female sub-populations selected under scenario 5

**Study population**	***p*value**	**Hazard ratio**	**95% confidence interval**	
			**Lower**	**Upper**
All	<0.001	2.468	2.205	2.762
Men	<0.001	2.418	2.131	2.745
Women	<0.001	2.681	2.098	3.426

**Figure 4:**
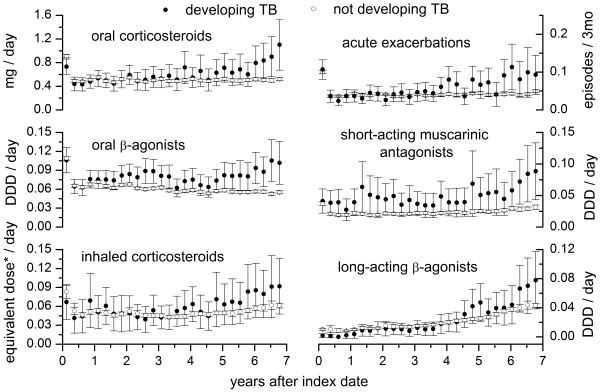
**Associated medication consumption trends.** The mean consumption (circle) and its 95% confidence interval (vertical error lines) aggregated every 90 days following the index date for nine medications among cases of chronic obstructive pulmonary disease selected under scenario 5. Oral and parenteral corticosteroids are presented as equivalent dose to prednisolone in milligrams, and the inhaled corticosteroids as equivalent dose to budesonide. Other medications are presented as defined daily dose (DDD).

Among the COPD cases selected under scenario 5, those who subsequently developed pulmonary TB were older (mean age 65.9 ± 11.4 years) and had a higher male–female ratio (3.5) than those not developing TB (Table 4). The mean interval between the index date of COPD and diagnosis of TB was 4.6 ± 2.6 years. During follow-up, the prescribed medications and number of AE episodes are presented in Figure 4. The COPD patients who subsequently developed TB received more oral β-agonists and short-acting muscarinic antagonists than those who did not develop TB, while the ICS dose was similar in the two groups. The number of AE episodes and consumption of OCS and LABA were initially similar in the two groups, but later increased more rapidly in those who subsequently developed TB.

**Table 4:** Characteristics of the COPD patients selected under scenario 5 at index date

	**Not developing TB (N = 22,920)**	**Developing TB (N = 674)**	***p *value**
Age (years)	54.2 ± 23.1	65.9 ± 11.4	<0.001*
Follow-up duration (years)	8.6 ± 2.5	4.6 ± 2.6	<0.001*
Male	14,110 (61.6)	525 (77.9)	<0.001^#^
Diabetes mellitus	1,566 (6.8)	55 (8.2)	0.179^#^
Malignancy	341 (1.5)	8 (1.2)	0.524^#^
lung cancer	36 (0.2)	1 (0.1)	>0.999^‡^
other cancer	305 (1.3)	7 (1.0)	0.513^#^
Autoimmune disease	68 (0.3)	2 (0.5)	>0.999^‡^
End-stage renal disease	35 (0.2)	2 (0.3)	0.285^‡^
Liver cirrhosis	8 (0.0)	0 (0)	>0.999^‡^
Pneumoconiosis	2 (0.0)	0 (0)	>0.999^‡^
Transplantation	4 (0.0)	0 (0)	>0.999^‡^
HIV/AIDS	0 (0)	0 (0)	
Low income	335 (1.5)	11 (1.6)	0.717^#^

HIV: human immunodeficiency virus, AIDS: acquired immunodeficiency syndrome; TB: tuberculosis;

Data are shown as mean ± SD or No. (%) unless otherwise indicated.

* calculated by the independent-samples *t* test.

^#^ calculated by the chi-square test.

^‡^ calculated by Fisher’s exact test.

**Figure 5:**
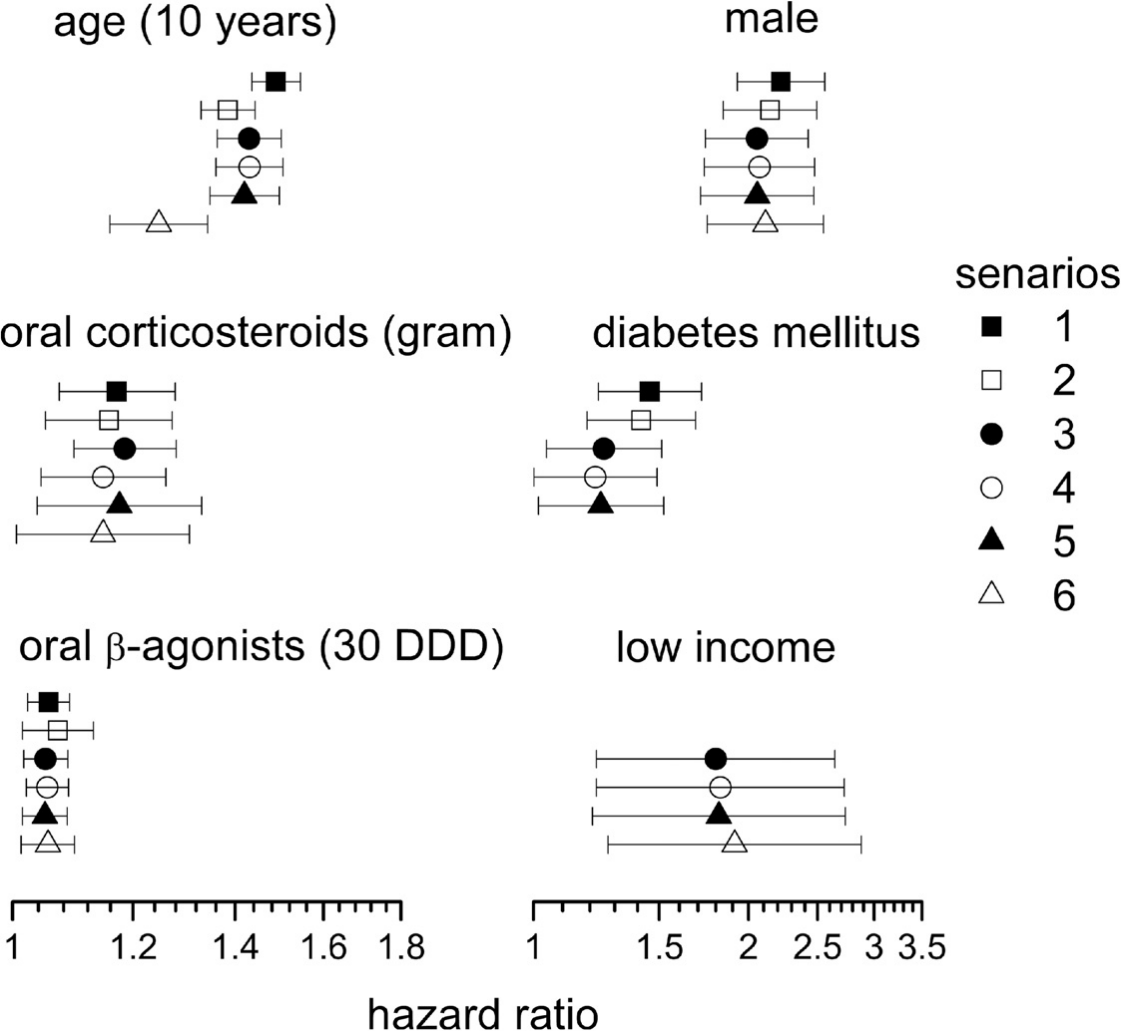
**Hazard ratios for developing tuberculosis.** Hazards ratio (marked circle) and its 95% confidence interval (horizontal error lines) of each independent risk factor for developing active tuberculosis (TB) among cases of chronic obstructive pulmonary disease (COPD) selected under different scenarios.

Univariate time-dependent Cox regression analysis for the risk of developing pulmonary TB in the 23,594 COPD patients selected by using scenario 5 revealed that age, male gender, diabetes mellitus, and low income status were risk factors. OCS, oral β-agonists and ICS dose-dependently increased the risk of developing pulmonary TB (Table 5). In multivariate analysis, however, the effect of ICS became obscured. Adding ICS into the multivariate model produced a minimal effect on the other co-variables. Although high-dose ICS was also significantly associated with pulmonary TB in univariate analysis, it was not an independent risk factor and had no effect on the other co-variables in multivariate analysis if using this categorical variable to replace the continuous variable – ICS. The independent risk factors for TB and their hazard ratios in the COPD patients selected under the six different scenarios were similar (Figure 5, Tables 6 and 7).

**Table 5:** Time-dependent Cox regression analysis for risk factors of pulmonary tuberculosis

**Variables**	**Univariate**		**Multivariate**		**Multivariate with ICS**	
	***p*value**	**HR (95% CI)**	***p*value**	**HR (95% CI)**	***p*value**	**HR (95% CI)**
Age (per 10 years)	<0.001	1.46 (1.38–1.54)	<0.001	1.42 (1.35–1.50)	<0.001	1.42 (1.35–1.50)
Male	<0.001	2.28 (1.90–2.73)	<0.001	2.06 (1.71–2.47)	<0.001	2.06 (1.71–2.47)
Diabetes mellitus	0.001	1.54 (1.26–1.88)	0.035	1.24 (1.02–1.52)	0.034	1.25 (1.02–1.53)
Oral corticosteroids^*^	<0.001	1.29 (1.15–1.44)	0.011	1.18 (1.04–1.33)	0.017	1.17 (1.03–1.32)
Oral β-agonists (per 30 DDD)	<0.001	1.06 (1.04–1.07)	0.005	1.05 (1.02–1.09)	0.006	1.05 (1.01–1.09)
ICS^#^	0.020	1.01 (1.00–1.02)			0.245	1.01 (0.99–1.03)
Low income	<0.001	2.04 (1.36–3.06)	0.004	1.82 (1.21–2.73)	0.004	1.82 (1.21–2.74)

HR: hazard ratio; DDD: defined daily dose; CI: confidence interval; ICS: inhaled corticosteroids. *Oral corticosteroids were converted to the equivalent dose of prednisolone 1 gram. ^#^ICS were converted to the equivalent dose of budesonide 24 mg.

Time-dependent Cox regression analysis for factors possibly associated with the development of active tuberculosis in cases of chronic obstructive pulmonary disease selected under scenario 5.

**Table 6:** Time-dependent Cox regression analysis for risk factors of pulmonary tuberculosis

**Variables**	**Scenario 1**		**Scenario 2**		**Scenario 3**	
	***p*value**	**HR (95% CI)**	***p*value**	**HR (95% CI)**	***p*value**	**HR (95% CI)**
Age (10 years)	<.0001	1.489 (1.436–1.545)	<.0001	1.385 (1.330–1.443)	<.0001	1.430 (1.363–1.501)
Male	<.0001	2.222 (1.929–2.559)	<.0001	2.145 (1.845–2.494)	<.0001	2.056 (1.741–2.427)
Diabetes mellitus	<.0001	1.456 (1.234–1.719)	<.0001	1.416 (1.189–1.687)	0.0163	1.256 (1.043–1.513)
Oral corticosteroids (g)	0.0004	1.171 (1.073–1.279)	0.0028	1.157 (1.051–1.273)	<.0001	1.185 (1.097–1.281)
Oral beta-agonists (30 DDD)	0.0007	1.056 (1.023–1.090)	0.0115	1.071 (1.015–1.130)	0.0029	1.051 (1.017–1.087)
Inhaled SAMA^*^ (30 DDD)	0.001	1.043 (1.017–1.069)	0.0053	1.039 (1.011–1.068)		
Acute exacerbations	0.0008	1.026 (1.011–1.042)	0.0127	1.021 (1.004–1.038)		
Low income					0.0027	1.8 (1.225–1.644)

HR: hazard ratio, CI: confidence interval, SAMA: short-acting muscarinic antagonists, DDD: defined daily dose

Time-dependent Cox regression analysis for the factors possibly associated with the development of active tuberculosis in cases of chronic obstructive pulmonary disease selected under scenarios 1–3.

**Table 7:** Time-dependent Cox regression analysis for risk factors of pulmonary tuberculosis

**Variables**	**Scenario 4**		**Scenario 5**		**Scenario 6**	
	***p*value**	**HR (95% CI)**	***p*value**	**HR (95% CI)**	***p*value**	**HR (95% CI)**
Age (10 years)	<.0001	1.431 (1.360–1.505)	<.0001	1.420 (1.348–1.497)	<.0001	1.248 (1.159–1.343)
Male	<.0001	2.073 (1.736–2.477)	<.0001	2.058 (1.714–2.471)	<.0001	2.113 (1.752–2.549)
Diabetes mellitus	0.0483	1.222 (1.001–1.490)	0.0349	1.243 (1.016–1.522)		
Oral corticosteroids (g)	0.0043	1.247 (1.044–1.261)	0.0107	1.176 (1.038–1.331)	0.0404	1.147 (1.006–1.307)
Oral beta-agonists (30 DDD)	0.0011	1.054 (1.021–1.088)	0.0046	1.050 (1.015–1.086)	0.009	1.055 (1.013–1.098)
Low income	0.0031	1.828 (1.226–2.725)	0.004	1.819 (1.210–1.734)	0.0018	1.914 (1.273–2.879)

HR: hazard ratio, CI: confidence interval, DDD: defined daily dose.

Time-dependent Cox regression analysis for the factors possibly associated with the development of active tuberculosis in cases of chronic obstructive pulmonary disease selected under scenarios 4–6.

## Discussion

The present study has three important contributions and findings. First, we found that COPD patients were more likely to develop pulmonary TB than non-COPD subjects under a wide variety of diagnostic scenarios for COPD (scenarios 1 to 6, Figure 1). Second, ICS was not a risk factor for developing active pulmonary TB among COPD patients after considering important clinical characteristics and other prescriptions. Third, COPD patients who received higher doses of oral corticosteroids and oral β-agonists were more likely to develop active pulmonary TB.

In univariate analysis, ICS dose-dependently increased the risk of developing pulmonary TB. Given that COPD is characterized by persistent and prominent airway inflammation and progressive airflow limitation [26,27], several randomized trials have demonstrated that ICS, with or without concomitant use of LABA, could improve pulmonary function and reduce AEs in patients with severe COPD [15,28], although it was associated with an increased risk of pneumonia [14,15]. Though the reported data are inconsistent, there is increasing evidence that long-term use of high-dose ICS may have systemic side effects such as cataracts, adrenal suppression, skin changes, and osteoporosis [29-31], and attenuate the adaptive immunity of the airways [32]. The latter complication can be more serious in TB-endemic areas, since suppression of the essential defense mechanism against *M. tuberculosis* in the airway and lung parenchyma may increase the risk of active pulmonary TB [16,17,33].

Systemic corticosteroids have been demonstrated to improve arterial hypoxemia and reduce the risk of treatment failure and length of hospital stay in AE of COPD [34,35]. In this situation, ICS alone may have a similar benefit while minimizing the side effects of systemic corticosteroids [36,37]. Therefore, ICS use in COPD patients may reduce the overall requirement of systemic corticosteroids by improving symptom control and preventing AE and hospitalization [15], resulting in a reduction in the risk of nosocomial exposure and the subsequent development of active TB. This probably explains the finding in the present study that the effect of ICS on the risk of developing pulmonary TB became insignificant when other factors, such as underlying comorbidities, OCS and oral medications were concomitantly considered in the multivariate model.

The finding that ICS use did not increase the risk of active TB in COPD patients is in contrast with a previous report from Taiwan [16] and the conclusion of a study using the insurance databases of Quebec [17]. In both studies, the average doses of OCS and ICS within a certain period of time were used to determine the intensity of drug exposure. Identification of COPD cases and the corresponding index dates in the Quebec studies depended on the prescription history of COPD medications. Therefore, their results may have been biased because drug usage usually varies with time, and a marked increase in drug usage may have been recorded at the initial COPD diagnosis which then fluctuated with the natural course of disease progression (similar to Figure 4). A better approach is to use a time-dependent covariate to represent the usage of each drug. In addition, although respiratory medications other than ICS and OCS have never been reported to increase the risk of TB, increased usage of these drugs as well as an increasing number of AE episodes may imply more severe COPD, requiring more frequent out-patient visits and hospitalization. These may increase the risk of infection by *Mycobacterium tuberculosis* and the subsequent development of active TB [38,39]. However, in the two studies, usage of respiratory medication and the number of AEs were not considered in the analysis.

The findings that age, male gender, diabetes, and receiving oral corticosteroids were risk factors for TB is consistent with the findings in prior literature [2,40,41]. Of note, the present study is the only cohort study conducted in a large general population controlled for a broad range of comorbidities, pharmacological and socioeconomic confounders in a time-dependent analysis. The frequency of AEs, which served as surrogate factors representing COPD severity and the risk of nosocomial TB exposure, remained significantly associated with the development of TB after adjusting for baseline clinical characteristics and the effect of corticosteroids in COPD patients selected under scenarios 1 and 2. However, the frequency of AEs became insignificant under scenarios 3 to 6, probably due to the reduced sample size. Furthermore, the association of increased use of oral beta-agonists and muscarinic antagonists to active TB suggests that the deterioration in pulmonary symptoms requiring increased medication could be a reflection of the development and progression of pulmonary TB. Therefore in TB endemic areas one should suspect TB when patients with chronic respiratory diseases deteriorate.

Smoking, the most important risk factor for COPD, has also been demonstrated to be associated with pulmonary TB [42]. Because the smoking status is not available in the NHIRD, it is possible that some of the effects of male gender on the development of pulmonary TB observed in present study are through the effect of smoking. However, if that were true, the hazard ratio of COPD for subsequent development of pulmonary TB should be higher in male sub-population than that in female counterpart since the prevalence of smoking was much lower among female COPD patients (24.2%) than that among general COPD patients (82.4%) in Taiwan [24,25]. The findings that COPD has a similar impact on the development of pulmonary TB in both genders suggest that the effect of COPD on the risk of pulmonary TB observed in the present study should not be completely attributed to smoking.

There are several limitations to this study. First, mycobacteriological results, pulmonary function tests and radiological findings were not available. Therefore, it is impossible to confirm the diagnosis and determine the severity of pulmonary TB and COPD. However, by using different scenarios to select COPD cases, our analysis showed that the identified risk factors for TB were very similar, if not completely the same, in a large and diverse population. We believe our conclusions can be applied to the management of COPD since the findings were generated from a national insurance database, which is a record of medical practice in the real world. Second, because COPD patients in Taiwan have a high prevalence of pulmonary colonization or infection by non-tuberculous mycobacteria (NTM), a very long duration of anti-TB treatment was chosen as one of the diagnostic criteria for pulmonary TB in the present study to exclude patients who received empiric treatment for TB and was later proven as NTM or other pulmonary diseases. However, by applying the very strict definition of TB, the incidence of active TB may have been underestimated because of default and early mortality. Third, the interpretation of the causal relationship between COPD and TB based on their temporality is difficult because COPD may be a complication of TB [43,44]. However, this may not be a serious concern because the trend of an increasing risk of TB persisted even after 10 years (Figure 3). Lastly, the estimation of drug exposure may have been biased since information on drug adherence was not available in the national insurance database. Further large-scale prospective studies are needed to address these issues.

## Conclusion

COPD patients are at high risk of developing pulmonary TB, especially those frequently receiving oral corticosteroids and oral β-agonists. Although ICS therapy has been shown to predispose COPD patients to pneumonia in large randomized clinical trials, it does not increase the risk of TB in real world practice. The selection criteria for COPD and TB defined in the present study may be helpful in further research using health insurance databases.

## Abbreviations

AE: Acute exacerbation; AIDS: Acquired immunodeficiency syndrome; COPD: Chronic obstructive pulmonary disease; HIV: Human immunodeficiency virus; ICS: Inhaled corticosteroids; LABA: Long-acting β-agonists; LHID: Longitudinal Health Insurance Database; NHI: National Health Insurance; OCS: Oral corticosteroids

## Competing interest

All authors have completed the Unified Competing Interest form at http://www.icmje.org/coi_disclosure.pdf (available on request from the corresponding author) and declare that (1) none have support from any company for the submitted work; (2) none have any relationships with any company that might have an interest in the submitted work in the previous 3 years; (3) their spouses, partners, or children have no financial relationships that may be relevant to the submitted work; and (4) none have non-financial interests that may be relevant to the submitted work.

## Authors’ contributions

Dr. Chih-Hsin Lee and Jann-Yuan Wang designed the study. Dr. Chih-Hsin Lee, Jann-Yuan Wang, Chor-Shen Lim, Ming-Chia Lee and Prof. Kun-Mao Chao were all involved in writing the manuscript and data interpretation. Ming-Chia Lee, Dr. Chin-Chung Shu, and Dr. Jann-Yuan Wang were involved in the statistical analysis. Dr. Chih-Hsin Lee is the guarantor for the manuscript. Prof. Li-Na Lee was the director responsible for general organization and instruction. All authors read and approved the final manuscript.

## Pre-publication history

The pre-publication history for this paper can be accessed here:

http://www.biomedcentral.com/1471-2334/13/194/prepub
